# Retraction: Thioredoxin Glutathione Reductase as a Novel Drug Target: Evidence from *Schistosoma japonicum*

**DOI:** 10.1371/journal.pone.0346111

**Published:** 2026-03-31

**Authors:** The PLOS One Editors

Following the publication of this article [1] concerns were raised about similarity with content in previously published articles [2–4] by the same group. The *PLOS One* Editors followed up with the authors and corresponding author CXY indicated that the following article content was previously published:

- The expression of recombinant *Schistosoma japonicum* thioredoxin glutathione reductase (SjTGR) was reported in [2].
- The reported thioredoxin reductase (TrxR), glutathione reductase (GR) and glutaredoxin (Grx) activities of the recombinant SjTGR in Table 1 of [1] are the same as those in Table 1 of [2].
- Fig 1 in [1] was previously published as Figure 1 in [2].
- Fig 2 in [1] was previously published as the left panel in Figure 2 in [2].
- Fig 3 in [1] was previously published as Figure 3 in [2].
- Fig 4 in [1] was previously published as the right panel in Figure 2 in [2], and as Figure 1 in [3].
- Fig 5 in [1] was previously published as Figure 4 in [2].
- Figs 6A-C in [1] were previously published as Figures 2-4 in [3].
- Figs 7A-C in [1] were previously published as Figure 1-3 in [4].
- Tables 1-3 in [1] report the same results as Tables 1-3 in [3].
- The paragraph titled “Toxicity of auranofin in cultured *S. japonicum* adult worms” in the Results section of [1] reports the same results previously described in [4].
- Articles [2–4] were not cited in [1].

Corresponding author CXY stated that [3] discusses the enzymatic characteristics of the recombinant TGR protein; [2–4] report a comprehensive study on the thioredoxin glutathione reductase of *Schistosoma japonicum* (SjTGR), including gene cloning, construction of selenium protein expression vector, expression and purification of recombinant SjTGR protein, enzymatic characteristic analysis, inhibitor screening *in vitro* and therapeutic experiments *in vivo*; and [1] combines the studies reported in [2–4]. Corresponding author CXY also stated that Table 4 reporting reduction of worm and egg numbers *in vivo* following auranofin treatment were not reported prior to the publication of [1].

Corresponding author CXY apologizes for the issues with the published article [1] and stated that the dual publication arose owing to CXY’s unfamiliarity with the rules of international journal publication at that time. Authors CXY, ZCH, and JHL stated that ZCH and JHL were not aware of the publication of [3].

In light of the above concerns which call into question the compliance of [1] with *PLOS One*’s publication criteria that results reported have not been published elsewhere [5], the *PLOS One* Editors retract this article.

LJS, JHL, ZCH, and CXY agreed with the retraction. SYX, CYQ, JW, WZ, and XRY either did not respond directly or could not be reached.

The retracted article [1] was removed from the *PLOS One* website at the time of retraction, due to the similarities with [2–4]. The article’s Copyright statement was also updated at that time, and the removed contents are no longer offered under the Creative Commons Attribution License.
